# The Nation and Its Paralytics and Epileptics

**Published:** 1895-04-27

**Authors:** 


### The Hospital

AN INSTITUTIONAL JOURNAL OF

The Medical Sciences and Hospital Administration,

WITH

AN EXTRA NURSING SUPPLEMENT.

Vol. XVIII.—No. 448.] [April 27, 1895.

### The Nation and its Paralytics and Epileptics.

No more interesting, no more pathetic subject of study could be imagined, alike for the practical man and for the student of "the ills of humanity," than the subject of the epileptic and the paralysed person. Familiarity breeds contempt! and though no amount of familiarity can breed contempt for helplessness and hopelessness, except in the base, yet familiarity with the epileptic and the paralysed has bred in vast numbers of us that which is twin brother to contempt, namely, unregarding indifference.

"Things Seen are Greater than Things Heard" is the title of a small publication which Mr. Burford Rawlings, of the National Hospital for the Paralysed and Epileptic, in Queen's Square, has just issued, no doubt with the consent and approval of the many eminent persons, from the Queen downwards, who take an active interest in so unique an institution. What Mr. Burford Rawlings means is this, that if we could only see with our own eyes and realise vividly in our own consciousness the actual condition of the victims of epilepsy and paralysis in this country, we should recognise that it is a duty which is nothing less than national to render to them all that practical sympathy and all those humane services which the best knowledge and science of the times afford.

The epileptic and the paralysed are "seen." It would be equally true to say of many of them that they are not seen. They are visible to the eye, but they are not understood, at any rate so far as most epileptics are concerned; and that is the pity of it. The epileptic is a man and he is not a man. He has a brain which can perceive, and reason, and will. But that brain consists of many parts, and some of those parts produce an undue proportion of nerve energy, whilst other parts do not produce enough. The energy of those parts which produce too much escapes, so to speak, from the control of those which produce too little, and thus we have an uncontrollable lunatic within the body of a sane man. In ancient times the epileptic was believed to be "possessed with a devil." Nobody who has ever seen epilepsy in its more dreadful forms will wonder at the superstition. Of the paralysed, why speak more at length? To be paralysed in sight, blind, that is to say; or to be paralysed in hearing, deaf; either of these is a grievous affliction. But to be paralysed throughout, to be unable to feel, unable to walk like other men, unable to work, perhaps unable to speak, or see, or hear; to be cursed with all these disabilities and yet to be conscious and know it all, and to be vividly aware that our fellow creatures all around us are active, busy, full of energy, and of every variety of work and pleasure, this surely is a doom to which the grave itself were infinitely to be preferred.

The nation, we say, being a humane nation, being a religious nation, owes nothing less to its paralysed and its epileptics than this, that it should take care of them and put them in the brightest and happiest places upon which the sun of modern science shines. To some extent this is done; and we, at any rate, are proud and thankful to know it. Mr. Burford Rawlings' little pamphlet will let everybody else know it who will take the trouble even to glance at its pictures. It is an honourable thing that the metropolis, in this eminently national cause, should have been so truly metropolitan and national as to build one hospital, not for London, or even for London and its suburbs, but for the whole nation.

That the nation should possess at least one hospital in which cerebral science is fully competent is manifestly indispensable if we would preserve our reputation for national humanity. That reputation we must sustain; it were degradation to think otherwise. That reputation, in this particular department, costs the nation £15,000 a year. "The nation," we have said; but it is to be feared that it is not the nation which finds the fifteen thousand a year. The nation, Mr. Rawlings tells us, finds many patients, and sends them up from England, Scotland, Ireland, Wales, the Channel Islands, and the Isle of Man. But the nation leaves London to pay the bill. John Bull is not seen at his best here.

There is some compensation for those metropolitan physicians and philanthropists who provide science and care for the nation's epileptics and paralytics in the fact that Her Majesty the Queen, the Archbishop of Canterbury, the late Sir Andrew Clark, Mr. Jonathan Hutchinson, F.R.S., and many others of the most distinguished representatives of the political greatness and of the religion and science of the times have striven to stir up the conscience of the nation on behalf of this peculiarly helpless and afflicted class. Our words to-day are not addressed to philanthropists only, but to men of science and every other class; and our ardent wish is that they may recognise, not only what science is doing for the victims of epilepsy and paralysis, but also, through the means of these very victims, what knowledge and practical skill she may yet acquire for the service of the whole nation. But for this, science must be provided with national resources; and it is for every man in the nation to recognise his own peculiar duty, and to provide a just proportion of those resources. The whole nation's work should be paid for out of the whole nation's pocket.

